# Genetic background-dependent abnormalities of the enteric nervous system and intestinal function in Kif26a-deficient mice

**DOI:** 10.1038/s41598-021-82785-1

**Published:** 2021-02-04

**Authors:** Yukiko Ohara, Lisa Fujimura, Akemi Sakamoto, Youichi Teratake, Shuichi Hiraoka, Haruhiko Koseki, Takeshi Saito, Keita Terui, Tetsuya Mitsunaga, Mitsuyuki Nakata, Hideo Yoshida, Masahiko Hatano

**Affiliations:** 1 Department of Pediatric Surgery, Graduate School of Medicine, Chiba University, Chiba, Japan; 2 Biomedical Research Center, Chiba University, Chiba, Japan; 3 Department of Biomedical Science, Graduate School of Medicine, Chiba University, 1-8-1 Inohana, Chuoku, Chiba City, Chiba 260-8670 Japan; 4 Laboratory for Developmental Genetics, RIKEN Center for Integrative Medical Sciences (RIKEN-IMS), Yokohama, Japan; 5 Department of Cellular and Molecular Medicine, Graduate School of Medicine, Chiba University, Chiba, Japan; 6 Department of Pediatric Surgery, Chiba Children’s Hospital, Chiba, Japan

**Keywords:** Animal disease models, Disease model, Animal breeding

## Abstract

The Kif26a protein-coding gene has been identified as a negative regulator of the GDNF-Ret signaling pathway in enteric neurons. The aim of this study was to investigate the influence of genetic background on the phenotype of Kif26a-deficient (KO, −/−) mice. KO mice with both C57BL/6 and BALB/c genetic backgrounds were established. Survival rates and megacolon development were compared between these two strains of KO mice. Functional bowel assessments and enteric neuron histopathology were performed in the deficient mice. KO mice with the BALB/c genetic background survived more than 400 days without evidence of megacolon, while all C57BL/6 KO mice developed megacolon and died within 30 days. Local enteric neuron hyperplasia in the colon and functional bowel abnormalities were observed in BALB/c KO mice. These results indicated that megacolon and enteric neuron hyperplasia in KO mice are influenced by the genetic background. BALB/c KO mice may represent a viable model for functional gastrointestinal diseases such as chronic constipation, facilitating studies on the underlying mechanisms and providing a foundation for the development of treatments.

## Introduction

The enteric nervous system (ENS) develops from neural crest precursor cells during embryogenesis. Enteric neural crest precursor cells colonize the entire length of the gut by migrating and proliferating along the long axis of the gastrointestinal (GI) tract. Most ENS precursor cells forms the myenteric ganglia within the muscularis externa, while a subset migrates to the inner submucosal layer and forms the submucosal ganglia^1,2^. Many important regulatory factors in this process have been identified, and genetic mutations associated with some of these factors are responsible for the pathogenesis of congenital aganglionic megacolon in mice and humans. In humans, signaling required for proper ENS development is derived from glial-line derived neurotrophic factor (GDNF) and its receptor RET^3–7^ and endothelin-3 and its receptor endothelin receptor type B^8–12^ as well as transcription factors such as SOX10^13,14^ and PHOX2B^15,16^. Furthermore, mutations in these genes have been reported in Hirschsprung disease. Intestinal neuronal dysplasia (IND) is an enteric neuropathy which shares phenotypic features with Hirschsprung disease. Numerous clinical signs and symptoms of IND such as abdominal distension, constipation, and megacolon are also observed in Hirschsprung disease. However, the pathology of IND is different from that of Hirschsprung disease. Hyperganglionosis, increased acetylcholinesterase activity, and ectopic ganglionic cells are characteristics of IND^17,18^. *Ncx* (also known as *Tlx2* or *Hox11L1*)-deficient mice develop hyperganglionic megacolon and represent a murine genetic model of IND^19,20^. Despite intensive study, no *HOX11L1*/*NCX*/*TLX2* gene mutations or candidate genes have been identified in humans^21^. Furthermore, penetrance of the megacolon phenotype in the deficient murine model is specific to the genetic background and indicates the presence of genetic modifiers^22^.

Kinesin superfamily proteins (KIFs) have been shown to transport membranous organelles and protein complexes through microtubules in an ATP-dependent manner. In total, 45 murine and human KIFs have been identified and classified into 14 large protein families identified as kinesin-1 through kinesin-14. Kif26a is a murine Kif belonging to the N-11 kinesins group^23^. Kif26a has a divergent motor domain that exhibits microtubule-binding activity but lacks ATPase activity, indicating functions other than cargo transport.

Indeed, Kif26a KO C57BL/6 mice developed a megacolon with enteric neuronal hyperplasia and died within 30 days of birth. Kif26a was found to be involved in the regulation of GDNF-Ret signaling by suppressing GDNF-Ret signaling through direct association with Grb2. This represents an important signal transduction pathway between Ret receptor tyrosine kinase and downstream molecules such as Akt and ERK^24^.

We have established a KO mouse colony with a BALB/c genetic background. In contrast to a previous report^24^, BALB/c KO mice did not develop a megacolon and survived for > 400 days. In this study, we aimed to assess GI tract function and the histopathology of enteric neurons in KO mice with both BALB/c and C57BL/6 genetic backgrounds. We demonstrate and discuss the phenotypic variations in Kif26a KO mice of two distinct genetic backgrounds.

## Results

### KO mice of the C57BL/6 genetic background died within 1 month; however, BALB/c mice survive for > 1 year

**Figure 1:**
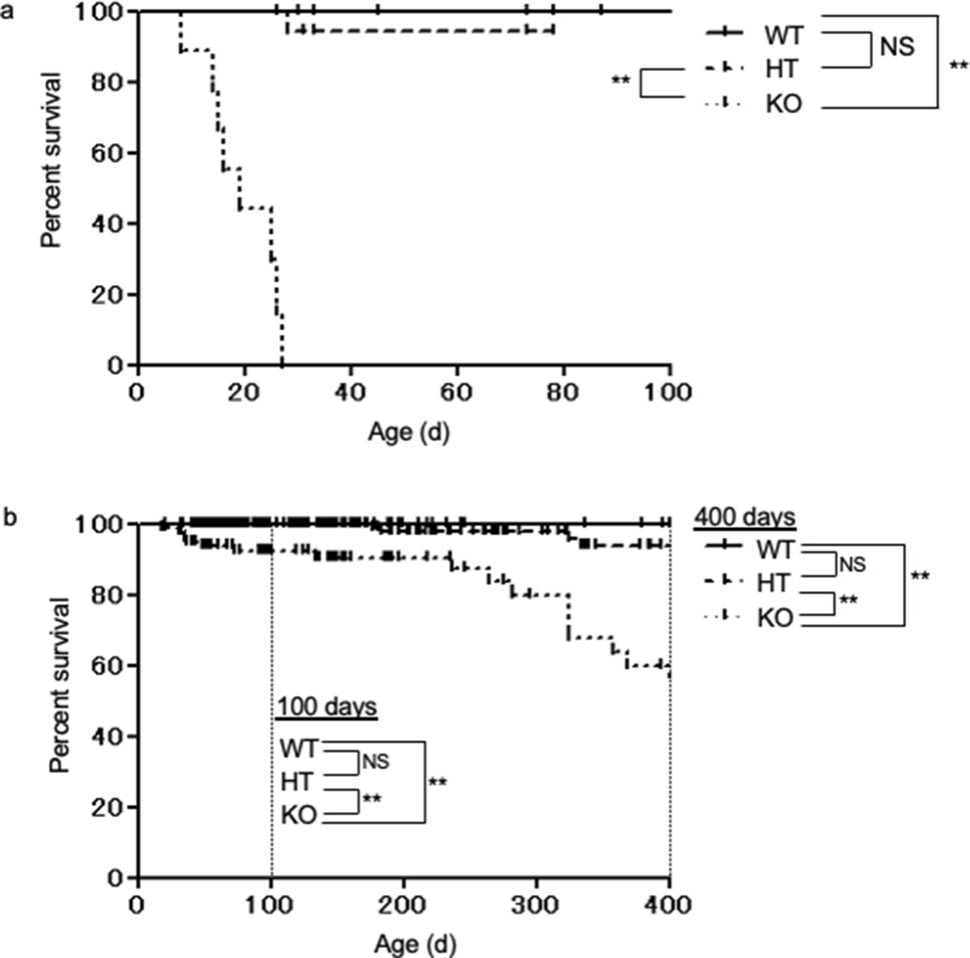
Survival curve for Kif26a-deficient (KO), heterozygous (HT), and wild type (WT) littermate mice of two different genetic backgrounds. (**a**) Survival curve of Kif26a mutant mice generated from C57BL/6 genetic background. WT (n = 27), HT (n = 17) and KO (n = 9) mice were observed up to 100 days. The median lifespan of the KO mice is the 19 days. The survival period of the KO mice is significantly shorter than WT and HT mice. (**b**) Survival curve of Kif26a mutant mice generated from the BALB/c genetic background. WT (n = 175), HT (n = 249), and KO (n = 98) mice were observed for more than 400 days. The median lifespan of the KO mice is 412 days. The survival period of KO mice is significantly shorter than that of WT and HT mice. The survival rate of KO mice was significantly lower than that of WT and HT mice, being 400 days. Furthermore, the survival rate of KO mice is significantly lower than that of WT and HT mice at 100 days (both p < 0.0001, log-rank test). *P < 0.05, **P < 0.01, *NS* not significant.

A previous study reported that KO mice developed megacolon with enteric neuron hyperplasia and subsequently died within 1 month^24^. We introduced KO mice to our animal facility and established C57BL/6 and BALB/c colonies. Kif26a heterozygous (HT) mice were backcrossed with C57BL/6 or BALB/c mice for nine generations and HT mice were interbred to obtain KO mice. As shown in Fig. 1a, KO mice with the C57BL/6 genetic background died within 30 days postpartum. The median lifespan of the C57BL/6 KO mice in our colony was 19 days. In agreement with a previous report^24^, autopsy revealed that the C57BL/6 KO mice developed megacolon. In contrast, approximately 60% of the deficient mice with the BALB/c genetic background survived for all 400 days of observation, and the median lifespan of the BALB/c KO mice was 412 days, although the survival rate of KO mice was significantly lower than that of WT and HT mice at 400 days (Fig. 1b). Furthermore, the survival rate of BALB/c KO mice was significantly lower than that of WT and HT mice at 100 days (both p < 0.0001, log-rank test). With the exception of slightly retarded growth in the BALB/c KO mice compared with WT mice, no other obvious abnormalities were noted. Upon autopsy, no megacolon or macroscopic abnormalities were observed in the BALB/c KO mice. These results indicate that the genetic background affects the phenotype of KO mice.

### Functional bowel abnormalities in BALB/c Kif26a-KO mice

**Figure 2:**
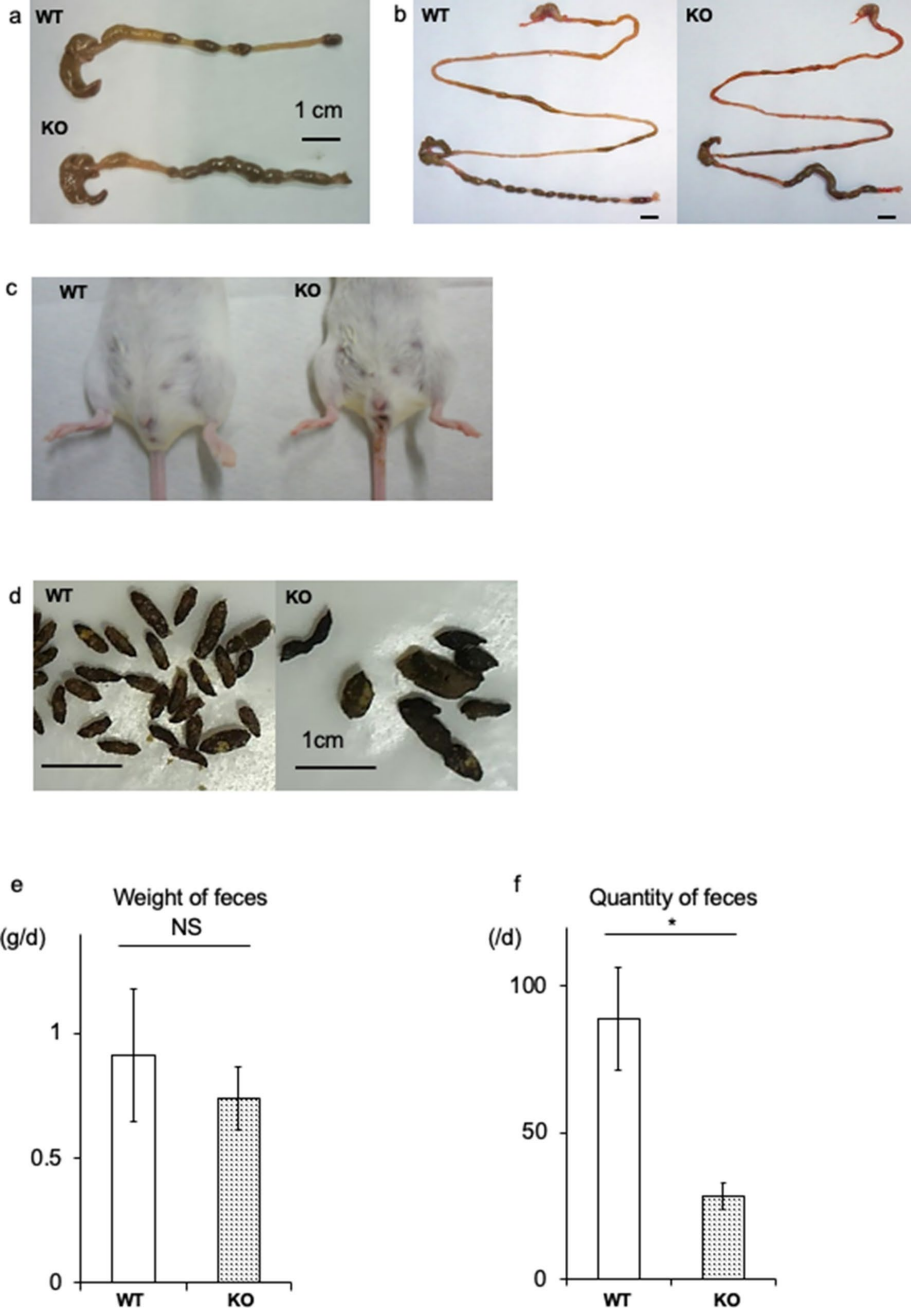
Functional analysis of the gastrointestinal (GI) tract. (**a**) Large intestine dissected from WT and KO littermates at 20 days. (**b**) Entire GI tract dissected from WT and KO mice. Note the delayed stool located in the middle segment of the large intestine in the KO mouse. (**c**) Anal region of WT and KO mice with watery stool attached around the anus in the KO mouse. (**d**) Fecal characterization. Feces of WT mice are large in number and similar in size. In contrast, feces of KO mice are small in number and irregular in size. (**e**,**f**) Comparison of the weight and quantity of daily feces. Total fecal weight expelled by KO mice is similar; however, the quantity is significantly less than observed in WT mice. This corresponds to higher weight per grain of feces. *P < 0.05, *NS* not significant.

In contrast to C57BL/6 KO mice, we did not observe megacolon in the BALB/c KO mice. One of the characteristic findings in BALB/c KO mice was fecal filling of approximately two-thirds of the colon, while distinct fecal masses were observed in WT mice (Fig. 2a,b). Around the anal regions, watery stool was observed in most of the KO mice (Fig. 2c). In contrast, the upper GI tract appeared similar to that of control mice (Fig. 2b). In the KO mice, the size of the feces was both heterogeneous and larger than that in the control mice (Fig. 2d); however, the fecal weight and quantity were lower (Fig. 2e,f). These findings indicate functional abnormalities in the GI tract of the BALB/c KO mice.

**Figure 3:**
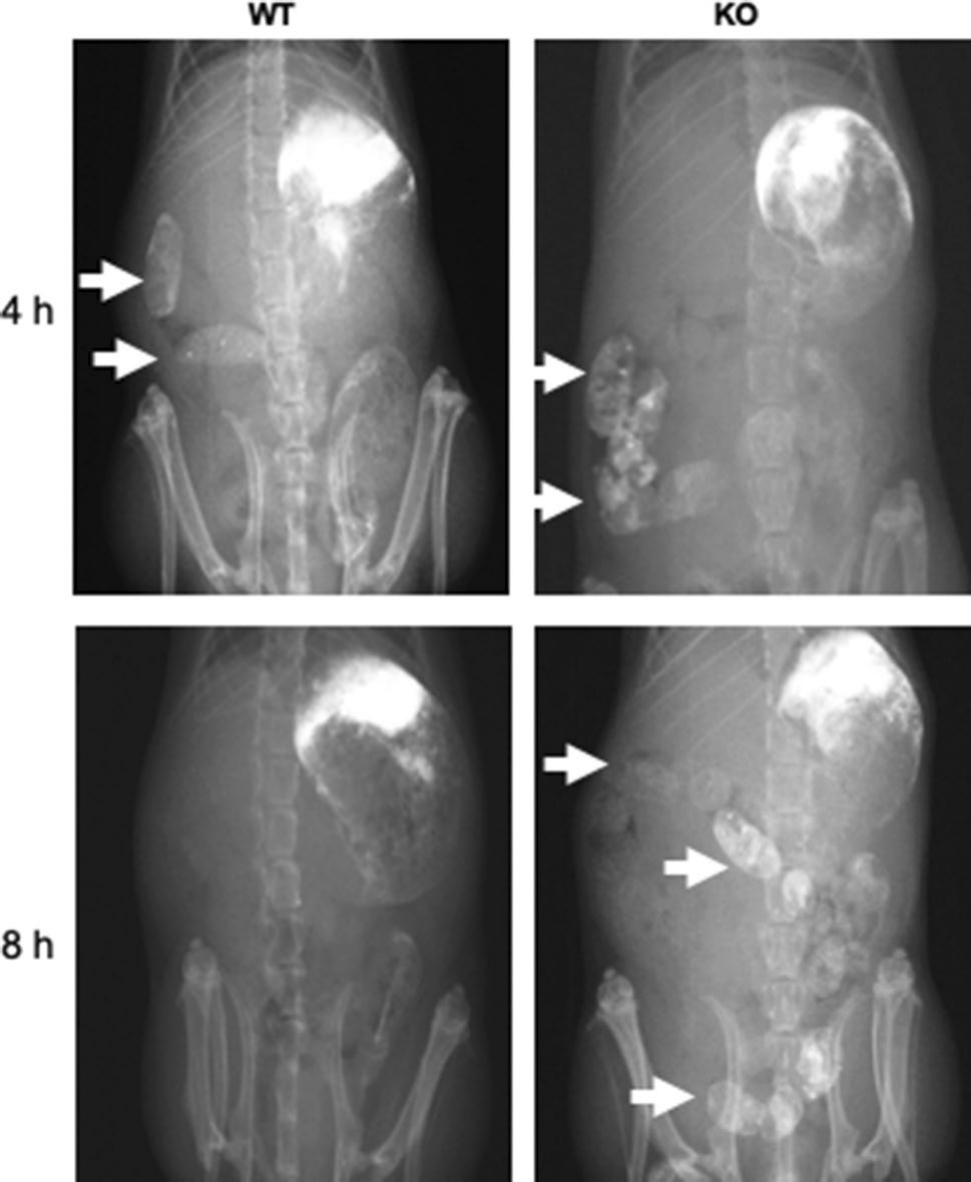
Intestinal transit time of the BALB/c Kif26a-KO mice. Barium sulfate was administered orally and radiographs obtained after 4 and 8 h. Barium is observed in the large intestine of the WT (n = 4, 20-week-old female), and KO (n = 4, 20-week-old female) mice at 4 h. After 8 h, barium sulfate remains in the large intestine (arrows) of the KO mice but has been excreted from the large intestine in the WT mice. Areas in the upper left part of each radiograph are associated with barium sulfate that remained attached to the stomach lining.

Furthermore, functional abnormalities of the GI tract were evaluated on the basis of barium transit time. Barium sulfate was administered to the stomach via a catheter, and the transit time was measured radiographically. As shown in Fig. 3, barium was excreted from the colon within 8 h after administration in wild type (WT) mice (Fig. 3: WT, arrows). In contrast, barium sulfate remained in the colon of the KO mice 8 h after administration (Fig. 3: KO, arrows). These data suggest that movement of fecal matter in BALB/c KO mice was impaired in the colon.

### Regional increase in enteric neurons in the BALB/c KO mice

Since C57BL/6 Kif26a KO mice exhibit enteric neuronal hyperplasia, we examined the population of these neurons in the BALB/c KO mice via in situ NADPH-diaphorase (NADPH-d) staining at 4 days and 7 weeks of age. Excised colons were incised longitudinally and cut into several segments. NADPH-d-positive enteric neurons were enumerated in each segment.

**Figure 4:**
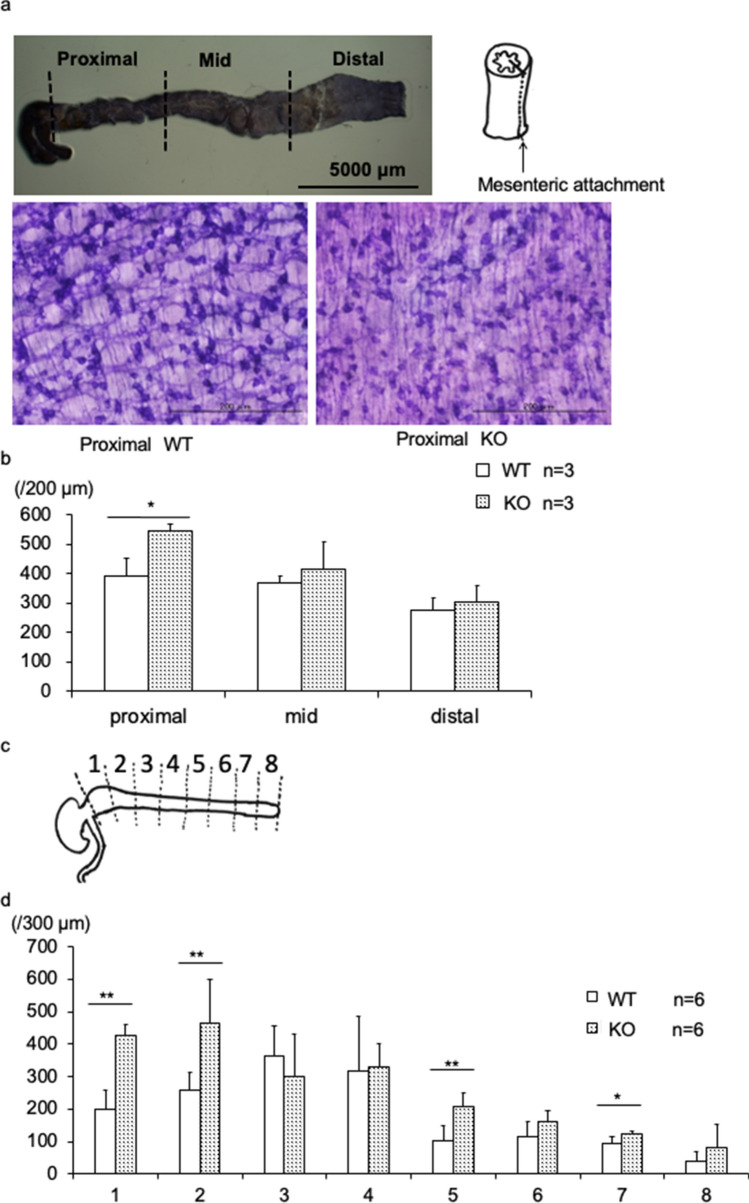
Number of NADPH-diaphorase (NADPH-d) positive enteric neurons in BALB/c Kif26a-KO mice. (**a**) Sample preparation from 4-day-old mice. The colon was divided into three parts (proximal, middle, and distal) and incised along a line following the points of mesenteric attachment. (**b**) Number of NADPH-d positive neurons in 200-μm-wide sections of proximal, middle, and distal colon from 4-day-old WT (open bars) and KO mice. (**c**) Sample preparations from 7-week-old mice. The colon was divided into eight parts and incised along the points of mesenteric attachment. (**d**) Number of NADPH-d positive neurons in 300-μm-wide section of colon divided into eight portions from 7-week-old WT (open bars) and KO mice.

For samples retrieved on day 4, specimens were divided into proximal, middle, and distal portions (Fig. 4a). A higher number of NADPH-d-positive enteric neurons were observed in the proximal colon of BALB/c KO mice. There were no differences in the number of enteric neurons in the middle and distal portion of the colon (Fig. 4b).

**Figure 5:**
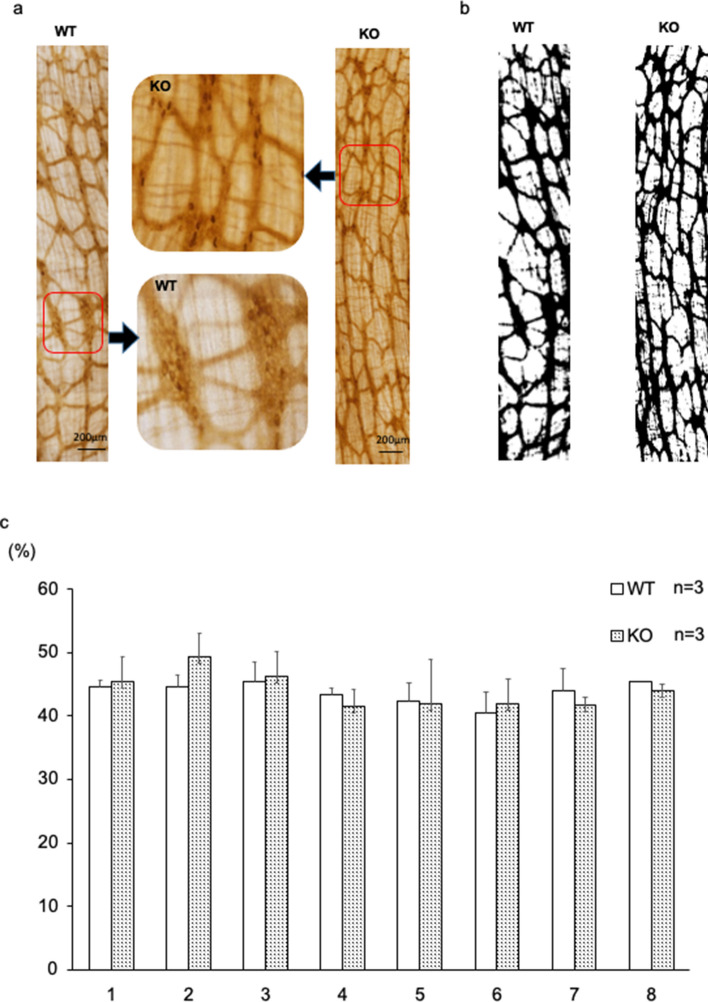
Area of acetylcholinesterase-positive enteric neurons in BALB/c Kif26a-KO mice. (**a**) Images of acetylcholinesterase-positive neurons in the most proximal part of the colon in 7-week-old WT and KO mice. A high-magnification view of the area demarcated in the middle panel. (**b**) Binarized image using ImageJ software. (**c**) Area percent of acetylcholinesterase-positive nerve fibers for each segment, as determined using ImageJ software. No significant difference was observed.

To confirm these results, we divided the colon equally into eight segments and enumerated the NADPH-d positive enteric neurons in 7-week-old mice (Fig. 4c). In the most proximal region of the colon, the number of NADPH-d positive enteric neurons doubled in the BALB/c KO mice compared to those in WT mice (Fig. 4d). In more distal segments of the colon, there was a statistically significant increase in the number of NADPH-d-positive neurons in the BALB/c KO mice. Subsequently, the areas of nerve fibers positive for acetylcholinesterase staining were compared, yielding no significant difference (Fig. 5a-c). Nerve fiber hyperplasia in BALB/c KO mice was partially confined compared with that in C57BL/6 mice.

### In silico analysis of genetic polymorphisms between C57BL/6 and BALB/c mice

To investigate the phenotypic differences between C57BL/6 and BALB/c mice, we screened for genetic polymorphisms using the mouse genome database (MGI Mouse Genome Informatics: http://www.informatics.jax.org, Mouse Genomes Project—Wellcome Sanger Institute: https://www.sanger.ac.uk/sanger/Mouse_SnpViewer/rel-1505). Genes involved in enteric nervous system development or those associated with Hirschsprung disease were identified and genomic variations were screened within 10 kbp upstream and downstream of the genes (Table 1). No single nucleotide variations and indels were observed in BALB/c mice compared to C57BL/6 mice in the exons of *Ret*, *Gdnf*, *Edn3*, and *Ednrb*. On the contrary, numerous variations were observed between C57BL/6 and BALB/c mice in exons and in the introns of *Gfra1*, *Gfra2*, and *Ece1*.

**Table 1:** Genetic polymorphisms in C57BL/6 and BALB/c mice.

Gene	Chr	Position	Single nucleotide variations				Indels				Structural variations	
			Up	Exon	Intron	Down	Up	Exon	Intron	Down		
*Ret*	6	118149745–118199744	0	0	2	0	0	0	1	0	−	Gdnf-Ret pathway
*Gdnf*	15	7808048–7839580	0	0	1	0	0	0	1	0	−	Gdnf-Ret pathway
*Gfra1*	19	58233581–58457946	5	8*^1^	> 900	7	5	1*^8^	165	2	+	Gdnf-Ret pathway
*Gfra2*	14	70888107–70981840	21	12*^2^	> 500	8	2	7*^8^	126	3	+	Gdnf-Ret pathway
*Edn3*	2	174758619–174786042	1	0	2	0	0	0	0	0	−	Endothelin pathway
*Ednrb*	14	103812615–103846476	0	0	3	0	0	0	0	0	−	Endothelin pathway
*Ece1*	4	137860212–137967229	4	49*^3^	> 1000	29	2	4*^8^	178	6	+	Endothelin pathway
*Phox2b*	5	67092397–67101301	6	6*^4^	0	9	4	2*^8^	4	4	−	Transcription factor
*Tlx2*	6	83066324–83072293	1	2*^5^	0	2	1	2*^8^	0	1	−	Transcription factor
*Kif26a*	12	112142135–112183747	0	3*^6^	1	0	0	0	0	0	−	Negative regulator of Ret
*Sox10*	15	79152908–79167240	9	12*^7^	27	15	1	4*^8^	5	1	−	Transcription factor

^*1^5′ UTR variant = 3, 3′ UTR variant = 3, missense variant = 2.

^*2^5′ UTR variant = 2, 3′ UTR variant = 6, synonymous variant = 4.

^*3^5′ UTR variant = 1, 3′ UTR variant = 28, synonymous variant = 19, missense variant = 1.

^*4^3′ UTR variant = 6.

^*5^3′ UTR variant = 1, synonymous variant = 1.

^*6^3′ UTR variant = 3.

^*7^3′ UTR variant = 8, synonymous variant = 4.

^*8^Indels in exon are located in the 5′ UTR or 3′UTR.

## Discussion

In this study, we established KO mice with a BALB/c genetic background. BALB/c KO mice did not develop megacolon and survived more than 400 days. Although no gross abnormalities were observed, some BALB/c KO mice died 40 days postpartum, and approximately 40% of the mice died 400 days postpartum. The exact causes of death were not clear and may be heterogeneous. Since Kif26a is expressed in not only enteric neurons but also other tissues such as the brain, lung, heart, and kidney, it may have diverse functions and other critical roles in these organs.

The number of enteric neurons increased only in the proximal segments of the colon in 4-day and 7-week-old BALB/c strain of KO mice. In addition, no proliferation of acetylcholine esterase-positive nerve fibers was observed in the BALB/c strain of KO mice. In contrast, the number of both types of enteric neurons increased throughout the entire colon in C57BL/6 KO mice.

The genetic background-specific phenotype in murine models of enteric neuron-related disorders such as Hirschsprung disease and Hirschsprung-related diseases has been reported^22,25–28^. *Ncx* (also known as *Tlx2* or *Hox11L1*) KO mice develop megacolon with enteric neuron hyperplasia and are considered a murine model of intestinal neuronal dysplasia^19,20^. All KO mice of the C57BL/6 background developed megacolon, and the penetrance of the phenotype was 15% among 129 strains^22^. Sox10^Dom^ mice are a model of Waardenburg-Shah syndrome, which is characterized by Hirschsprung disease and pigmentation abnormalities in the hair, skin, and eyes. Aganglionosis was more severe in mice with the C57BL/6 genetic background, while hypopigmentation was increased in those with the C3H background^25,26^. Furthermore, the *EdnrB* locus with C57BL/6 alleles was reported to be responsible for more severe aganglionosis in Sox10^Dom^ mutants^28^. Together, these previous reports and our data suggest that the C57BL/6 strain is susceptible to neural crest and enteric neuron-related disorders. Consequently, there may be genetic modifiers influencing neural crest derived cell development and associated disease phenotypes related to enteric neurons. Since genomic variations among mouse strains may be responsible for phenotypic differences between strains^29^, we compared sequence variations between C57BL/6 and BALB/c strains focusing on genes involved in enteric neuron development (Table 1). Large-scale sequence variations were identified in *Gfra1, Gfra2*, and *Ecel*, while limited variations were identified in *Ret, Gdnf, Edn3*, and *EdnrB*. Gfra1 and Gfra2 link GDNF and RET, and activate the GDNF/RET signaling pathway^30,31^. ECE1 enables EDN3 to activate the EDN3/EDNRB signaling pathway^31^. Since GDNF, RET, EDN3, and EDNRB themselves are molecules that form the basis of enteric neuronal development, there may be limited sequence variation between the two strains. In contrast, sequence variations such as single-nucleotide variations, indels, and structural variations in *Gfra1, Gfra2*, or *Ece1* may affect their expression and function, resulting in phenotypic differences of enteric neurons. Further investigation of such genetic modifiers through genetic analysis would help elucidate the complex molecular mechanism and pathophysiology of human Hirschsprung-related diseases and neurocristopathies.

In this study, local increases in NADPH-positive enteric neurons were mainly observed in the proximal colon. However, no difference was observed in the acetylcholinesterase-positive area in the colons of WT and KO mice, probably because of the difference in the experimental method. While acetylcholinesterase staining permits evaluation of the extent of aganglionosis and can identify differences in patterning of neural connectives between ganglia, detecting differences with this method in total number of neurons is not possible since the stain is not localized within neuronal soma as the NADPH-d method is. Although megacolon did not develop in the BALB/c KO mice, functional bowel abnormalities were detected. On autopsy, over half the length of the colon was filled with feces, and the colonic transit time was delayed, suggesting impaired colon motility. Fecal masses were large and heterogeneous in size, and the number of feces expelled per day was reduced. These signs indicate chronic constipation. Occasionally, the anal region of the KO mice was contaminated with attached watery stool, consistent with overflow incontinence. Chronic constipation is a functional gastrointestinal disorder associated with various factors such as abnormalities in enteric neurons, neurotransmitter, and dysbiosis. Genetic alterations in KIF26A may be a susceptible factor for chronic constipation in humans.

## Materials and methods

### Animals

Kif26a KO mice were generated and provided by Dr. H. Koseki (RIKEN, IMS, Yokohama, Japan). A targeting vector was constructed by replacing the genomic region containing exon 6–11 coding consensus motif of kinesin of Kif26a with the neomycin resistant cassette. The diphtheria toxin-A fragment cassette was inserted downstream of the short arm. The targeting vector was transfected into R1 ES cells (derived from 129/Sv) through electroporation (Bio-Rad) (Fig. S1). Kif26a HT (+/−) mice were backcrossed with WT (+/+) C57BL/6 or BALB/c inbred strains for a minimum of nine generations. HT mice were interbred to produce KO (−/−) mice. Mice were maintained at Chiba University under specific pathogen-free conditions at 23 ± 3 °C, 55 ± 15% humidity, and on a 12:12-h light/dark cycle (lights on from 7:00 to 19:00). Mouse genotypes were determined using the 5′-GTTGAGGATGCTCTTGTCTC-3′ upstream primer and the 5′-ACATTGATATGCAGAGCCTC-3′ downstream primer for the WT allele, and the 5′-CTCGTGCTTTACGGTATCG-3′ upstream primer and the 5′-TCTCACAAGACTCGTTTGAG-3′ downstream primer for the KO allele.

All experiments performed comply with Japanese legislation including Act on Welfare and Management of Animals (Act No. 105, 1973) and Act on the Conservation and Sustainable Use of Biological Diversity through Regulations on the use of Living Modified Organism (Act No. 97, 2003). All experimental procedures were approved by the Institutional Animal Care and Use Committee of Chiba University and were carried out in accordance with the National Institute of Health guidelines and the ARRIVE guidelines.

### Functional analysis of the GI tract

Intestinal clearance times were evaluated through barium sulfate administration. Animals (20 weeks old) were fasted for 12 h prior to barium administration. Barium sulfate (300 μl) was orally administered using a catheter. Radiographs were obtained 4 and 8 h after barium sulfate administration, and barium excretion was evaluated. To evaluate the transit time of the small intestine, mice (7 weeks old) were fasted for 19 h and orally administered an EBD solution as previously reported^32^. After cervical dislocation, the intestines were extracted 30 min after EBD administration, and the distance from the terminus of the stomach to the leading edge of the dye front was determined. The extent of EBD transport was divided by the total small intestinal length to determine the percentage of intestinal transit.

For fecal characterization, mice were isolated in metabolic cages (AS ONE Corporation, Osaka, Japan) for 24 h with ad libitum access to food and water. Feces were collected, weighed, and enumerated every 6 h.

### Histochemical analysis of NADPH-d

The colon was resected and soaked in fresh 4% paraformaldehyde fixative for 2 h at 4 °C. Fixative rinse was performed in PBS for 24 h at 4 °C. The colon was sectioned into short segments and incised along the line of mesenteric attachment. Using a stereomicroscope (Leica, Wetzlar, Germany), the mucosa of each colon segment was dissected out of the outer muscular layer and serosa. Thereafter, the remaining tissue was incubated and shaken in a reagent mixture consisting of 1.0 mg/ml β-NADPH-d (N7505, Sigma-Aldrich, St. Louis, MO, USA), 0.1 mg/ml nitroblue tetrazolium (N6876, Sigma-Aldrich), 0.3% TritonX-100, and 0.1 M phosphate buffer for 1 h at 37 °C. The reaction was terminated by rinsing the segments in PBS^33^.

Colon segments were assessed and photographed using a stereomicroscope (Leica). The enteric neurons identified in micrographs of 200-μm- or 400-μm-wide sections of the colon segments were counted without any identifying information regarding the sample genotype.

### Histochemical analysis of acetylcholinesterase

Colon segments were resected and soaked in saturated sodium sulfate at 4 °C. Whole-mount samples were cut and fixed in 4% paraformaldehyde fixative for 2 h at 4 °C. A fixative rinse was performed in PBS for 24 h at 4 °C. The segments were subsequently incubated and shaken in a reaction mixture of ethopropazine HCl (7.2 mg) (E5406, Sigma-Aldrich), acetylthiocholine iodide (115.6 mg) (A5751, Sigma-Aldrich), glycine (75 mg) (MP Biomedicals, Fountain Parkway Solon, OH, USA), cupric sulfate (50 mg) (NACALAI TESQUE, INC., Kyoto, Japan), sodium acetate (885 mg) (NACALAI TESQUE, INC., Kyoto, Japan) and double distilled water (100 ml) for 75 min at room temperature. Samples were rinsed six times in distilled water, and incubated for 1–2 min after adjusting the pH to 6.0 with 1.25% sodium sulfate^34^. After six additional rinses in distilled water, photographs were obtained for each sample using a stereomicroscope (Leica). We analyzed the fields of view with high nerve fiber density using ImageJ. Nerve fibers were distinguished from the background in accordance with a preset threshold value, and the black and white areas were determined. The threshold was set at a mean filter confirming the value at which the fibers could be identified.

### Statistical analysis

Survival curves were generated and analyzed using GraphPad Prism (GraphPad Software, La Jolla, CA, USA). Between-group comparisons were performed using a two-tailed Student’s *t*-test. Overall survival was calculated using the Kaplan–Meier method, and comparisons were evaluated using the long-rank test. Results with *P* < 0.05 were considered significant.

## Supplementary Information

Supplementary Information.

## Data Availability

The original data of the published figures is available by requesting to the correspondent author.
