# Supplementary material for: Genetic background-dependent abnormalities of the enteric nervous system and intestinal function in Kif26a-deficient mice
Source: Sci Rep. 2021 Feb 4;11:3191. doi: 10.1038/s41598-021-82785-1 (PMC7862435; doi:10.1038/s41598-021-82785-1)
Supplement: Supplementary file 1 — Supplementary Information. [file 41598_2021_82785_MOESM1_ESM.pdf]

Supplementary information

Title:

Genetic background-dependent abnormalities of the enteric nervous system and intestinal function in Kif26a-deficient mice

Yukiko Ohara, MD, PhD[1], Lisa Fujimura, PhD[2], Akemi Sakamoto, MD, PhD[2, 3], Youichi Teratake, PhD[2], Shuichi Hiraoka, PhD[4], Haruhiko Koseki, MD, PhD[4, 5], Takeshi Saito, MD, PhD[1], Keita Terui, MD, PhD[1], Tetsuya Mitsunaga, MD, PhD[6], Mitsuyuki Nakata, MD, PhD[1], Hideo Yoshida, MD, PhD[1] and Masahiko Hatano, MD, PhD[2,3]*

[1]Department of Pediatric Surgery, Chiba University, Graduate School of Medicine, Chiba, JAPAN

[2]Biomedical Research Center, Chiba University, Chiba, JAPAN

[3]Department of Biomedical Science, Chiba University, Graduate School of Medicine, Chiba, JAPAN

[4]Laboratory for Developmental Genetics, RIKEN Center for Integrative Medical Sciences (RIKEN-IMS), Yokohama, Japan

[5]Department of Cellular and Molecular Medicine, Chiba University, Graduate School of Medicine, Chiba, JAPAN

[6]Department of Pediatric Surgery, Chiba Children's Hospital, Chiba, JAPAN

*Correspondence: Masahiko Hatano, MD, PhD, Department of Biomedical Science, Chiba University, Graduate School of Medicine, 1-8-1 Inohana, Chuoku, Chiba City, Chiba, 260-8670, JAPAN

E-mail: hatanom@faculty.chiba-u.jp

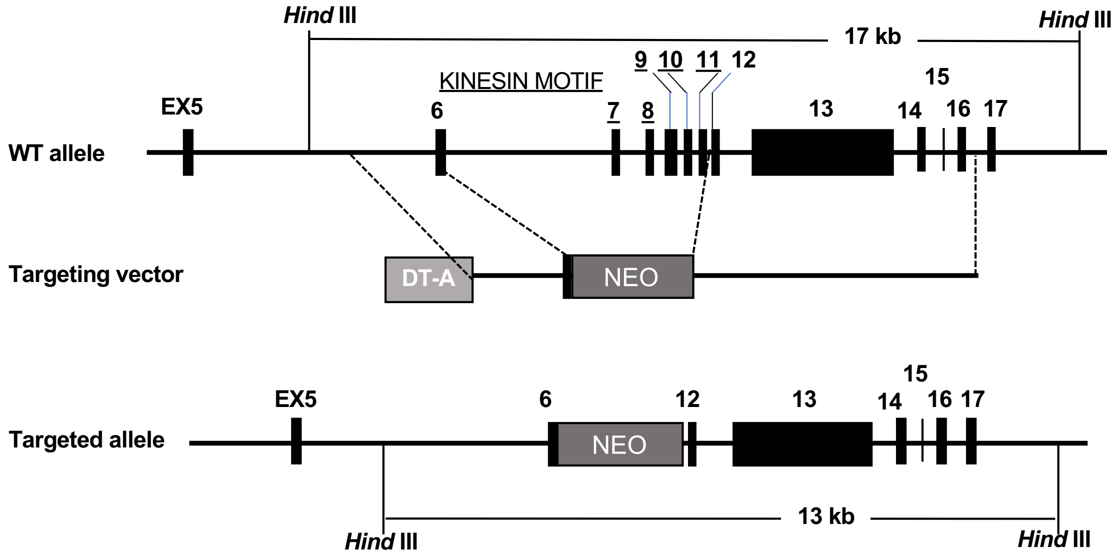

Figure S1: Targeted disruption of *Kif26a*

The Kif26a locus containing exons 5–17 is displayed on the top. The targeting vector displayed in the middle was designed to replace exon 6 with exon 11 coding a consensus sequence of the kinesin motif (underlined) of Kif26a with a neomycin resistance cassette (NEO). A diphtheria toxin A gene cassette (DT-A) was used for negative selection. The targeted allele is displayed in the bottom panel.
